# ‘HOPtimise’ Logic Model: An Organisational Innovation to Improve Nursing Practices in Paediatric Oncology in Québec

**DOI:** 10.1111/hex.70761

**Published:** 2026-07-14

**Authors:** Martin Blanc, Marie‐Pierre Gagnon, Daphney St‐Germain, Boutheina Mejri, Marie Gagné, Isabel Bean, Gratianne Vaisson

**Affiliations:** ^1^ CHU de Québec‐Université Laval Research Center Québec Quebec Canada; ^2^ Santé des Populations et Pratiques Optimales en Santé Québec Quebec Canada; ^3^ Faculty of Nursing Université Laval Québec Quebec Canada; ^4^ Reproduction Santé de la Mère et de l'Enfant Québec Québec Canada; ^5^ Nursing Service Québec Hospital – CHUL Québec Quebec Canada

**Keywords:** logic model, nursing practices, organisational innovation, paediatric oncology

## Abstract

**Introduction:**

HOPtimise is an organisational innovation in paediatric oncology in the province of Québec (Canada) destined to improve and reinforce best nursing practices through digital training using a serious game and create dashboards focused on indicators sensitive to the quality of care. This complex intervention is led by a quality improvement committee, a joint committee of user partners (former patients and family members) and healthcare providers. The present study aims to demonstrate how innovation and training are co‐created with multiple stakeholders. The involvement of patients and their families is at the core of the innovation's design. The aim of this study was to create and elaborate on the components of the logic model of the innovation in order to make it easily understandable for any stakeholder, to set the central roles and activities of the innovative process and to be able to verify the progress of the elaboration and the implementation of the innovation afterwards. The study also intended to generate guidance for future complex interventions in nursing practice improvement.

**Design:**

This study has a qualitative, descriptive and participatory design with a mixed data collection destined to create the logic model of the innovation. The qualitative methods used are organised through a three‐step timeline: the elaboration of the logic model, the verification phase and validation. Data collection methods involved two focus groups, each one at a different phase of the process (*n* = 9 and *n* = 6), semi‐structured interviews (*n* = 4) and verification checklists (*n* = 9) conducted from June 2022 to June 2024. Data collection guides are based on the Porteus (2009) method for the elaboration of a logic model. Through the process, *n* = 15 participants took part in at least one data collection (either focus group or interviews).

**Results:**

With a 2‐year and *n* = 15 multistakeholder study, the team obtained a logic model of organisational innovation. The HOPtimise logic model is a necessary tool to communicate with all stakeholders from various backgrounds. The study shows that the logic model was clear enough to all the participants (*n* = 15). As a result, this logic model is a strong base to stabilise the roles and activities of those stakeholders in the elaboration and implementation of the innovation. Indeed, it allowed the building of a stable quality improvement committee. Finally, the logic model became a baseline for the process of identifying barriers and implementation strategies.

**Conclusions:**

The HOPtimise logic model is representative of the complexity of the organisational innovation and the organisational challenges that the project will encounter, and is a necessary tool for evaluation of its implementation in terms of its co‐constructive nature.

**Patient and Public Contribution:**

This study is a co‐creation and is family‐centred. It involved the engagement and participation of user partners and organisational members. The research team interacted with them during the three steps of the elaboration of the logic model. They provided opinion and expertise on the content of the innovation, its components, the activities needed to reach the outcomes and implementation strategies.

**Abbreviations**
BCW: Behaviour Change Wheel
CCS: Canadian Cancer Society
CFIR: Consolidated Framework for Implementation Research
ERIC: Expert Recommendations for Implementing Change

## 1 Introduction

This study presents the logic model of the elaboration of a complex innovation named HOPtimise, an oncological paediatric program in the province of Quebec, Canada. This innovation aims to improve and reinforce best practices by co‐creating digital training for nurses using a serious game. The field of haematology and oncological paediatric is a highly specialised subject, and the field does not have enough innovation programs destined to improve nursing practices. Even if paediatric cancers account for 1% of all the diagnosed cancers in Canada [1], their impact on patients and their families is important.

The 5‐year survival ranges approximately from 80% in high‐income countries to 50% in upper‐middle‐income countries and to less than 30% in low‐ and medium‐income countries. This can be explained by poor access to cancer services [2, 3]. In Canada, this 5‐year rate of survival is up to 86% (McKenzie et al. 2025), and in the province of Québec, 84% of the diagnosed children survive at least 5 years [1].

Nurses represent the largest group of healthcare professionals and are the primary workforce caring for children with cancer [3]. For this reason, they are central to the complex intervention that is HOPtimise. However, paediatric oncology nurse training and official recognition of this specialisation are not universal, which makes delivery of safe and knowledgeable nursing care challenging [2]. Improvement is still needed [4], including the standardisation of best practices in paediatric oncology nursing training. Current nursing programs typically prepare graduates at a generalist level, despite the specialised nature of the field. Nurses must further specialise in specific clinical services and care after graduation [5, 6]. Most generic nursing programs devote only a few hours to oncology and, rarely to paediatric oncology (for instance, Université Laval offers a 3‐hour module in paediatric oncology). In Canada, one of the only nursing training programs in oncology (started in 1994) ended after a decade [5]. Despite increasing success in childhood cancer treatment, nursing practices in paediatric oncology still need support from health institutions and innovative programs to develop expertise. The standardisation of best practices remains an organisational challenge [7, 8]. Thus, organisational transformation is required, notably through the implementation of training programs and complex intervention based on multistakeholder consensus and involvement [9, 10]. For example, The SickKids Haematology/Oncology Nursing Program [5, 11] is a global initiative in paediatric cancer. Organised around a 10‐day course and for experienced nurses, this program, destined to improve nursing practices, is based on clinical mentorship, focusing on chemotherapy, central line care and psychosocial support. It is also family‐centred and collaborative. To date, no program is available in French, the official language in Québec.

Regarding HOPtimise, the novelty lies in the ongoing and complex involvement of user partners at every phase of the elaboration, implementation and evaluation of the intervention. To ensure the success of the program, the team co‐created a logic model with a range of stakeholders (experts, parents and survivors, and organisational actors) using a multimethod process [10]. A logic model is a visual support that describes the program and succinctly explains all components of the innovation and their relationship to the program's main objectives [12]. It also specifies the series of activities that draw on a set of resources and are designed to achieve specific results within target groups. It is an asset for planning and evaluating the implementation of an innovation and helps bridge the gap between strategic and operational planning (Porteus 2002). It coordinates research and evaluates all necessary activities by highlighting the program's outcomes. The logic model describes and illustrates ‘the content (what?), the recipients (who?), and the purpose (why?) of the program’ [13, p. 87], which are mandatory components. The content refers to the program's components related to the innovation's goals (a series of related activities) and the more specific activities that are carried out to meet these goals. The recipients are the target audience and people—individuals, groups or structures—meant to benefit from the implementation. The purpose refers to the outcomes that the team aims to achieve by implementing the innovation. There are optional components suggested by Porteus [13] in our elaboration of the logic model: inputs, outputs, contributing factors and external factors. Inputs are the resources enabling the activities and outputs are the services, products and/or events resulting from these activities. Short‐term outcomes refer to changes directly affecting the target population, whereas the medium‐term outcomes are broader consequences for oncological services, patients, doctors and others. Contributing factors explain why activities and outputs lead to results (including, e.g., the satisfaction of patient or of nurses regarding the programme). External factors are elements outside the program that impact results.

The specific aims of this study are as follows:

- to describe the co‐design iterative process that led a composite team and actors from different backgrounds to elaborate, verify and validate the logic model of HOPtimise;
- to demonstrate the causal relations between the goals and the elements of the program through collaborative development, and
- to show the different components of the logic model and the roles of the actors involved.

## 2 Methods

### 2.1 Context and Description of the Program

HOPtimise is an organisational innovation taking place at *Centre mère‐enfant Soleil*/*CHU de Québec‐Université Laval* (*CHUL*) that aims to improve nursing practices in the treatment of children with cancer in Quebec, by creating a serious game for nurses' training. The project integrates experiential knowledge from both organisational actors and families, and the expertise of healthcare providers to co‐design the innovation. The patients' experience is understood as expertise [14], and the importance of patient engagement in healthcare research and programs is increasingly recognised [14, 15].

The organisational innovation HOPtimise is composed of three main aspects: improvement of nursing practices in HOP, based on Michie's framework Behavior Change Wheel [16], through the creation of a serious game to help nurses reach professional excellence using digital training as part of the practice improvement [17, 18]; patient engagement in health innovation (based on Carman et al. [19] and Bombard et al. [20]); and building of indicator dashboards to measure the impact of the innovation. The project has two main dimensions: innovation and research.

The innovation involves the prioritisation of nursing practices to consolidate, in partnership with patients. The research team's role is to support the development of the innovation, to evaluate the results and assess the impacts of its implementation on care quality indicators and services in paediatric haemato‐oncology. HOPtimise is co‐created with patients and families, with managers and organisational actors, healthcare providers and researchers. The co‐creation of the logic model was based on the design thinking method inspired by the work of Porteus [13], and the innovation is strongly founded on patient engagement. The goal is to include patients and families in the organisational design and the governance [19] through the whole process. The user partners are co‐designers of the entire innovation and participate at each step of the elaboration of the innovation, its implementation and the assessment of the program. Furthermore, if the patients are involved in the elaboration of the logic model, it is easier to evaluate the continuum of patient engagement through the whole project and, afterwards, to evaluate the program itself [21] and its implementation [13].

### 2.2 Design of the Study

#### 2.2.1 Sampling and Participants

This study used a qualitative and descriptive design, and a participatory approach [12, 15, 21, 22, 23]. The team analysed data extracted from focus groups, interviews and checklists, and triangulated common themes to mitigate potential biases [24, 25]. The synthesis was visually represented through the main components of the logic model. The use of an elaboration diary reinforced the study's validity [26, 27], facilitating communication between the research team as analysts and the data. The diary entailed recording of every activity of each member involved in the elaboration of the innovation process and the logic model, creating a chronological record [26, 27]. This process ensured that the logic model's elaboration met criteria established in the literature [12, 15, 22].

Participants in the study were stakeholders involved in the co‐creation of the logic model. These individuals participated in one or more of the three steps of the logic model.

As can be seen in Table 1, throughout the process, 15 stakeholders participated in the elaboration, verification and validation of the logic model. For comparison, Balice‐Bourgois et al. [10] involved 16 participants to reach expert consensus for the intervention's development, and Bisson et al. [15] used a sample of 22 to create a logic model in three steps. Additionally, nine co‐researchers served as advisers and were available for consultation during the process.

**Table 1:** Participants involved in one or several phases of the co‐creation of HOPtimise logic model.

Status as a stakeholder	Professional background/additional information	*N* = *x*	Total *n* = *x*
Co‐lead researcher	Nurse‐PhD	1	2
	Physician, head of HOP department	1	
Researchers	Research assistants and professionals	4	4
Organisational and strategic actors	Advanced practice nurses	2	5
	Physician	1	
	Nurses—executive status in the service	2	
Users partners	Parents of children who were cancer survivors	3	3
Expert user partner	Cancer survivor	1	1
			*N* = 15

**Figure 1:**
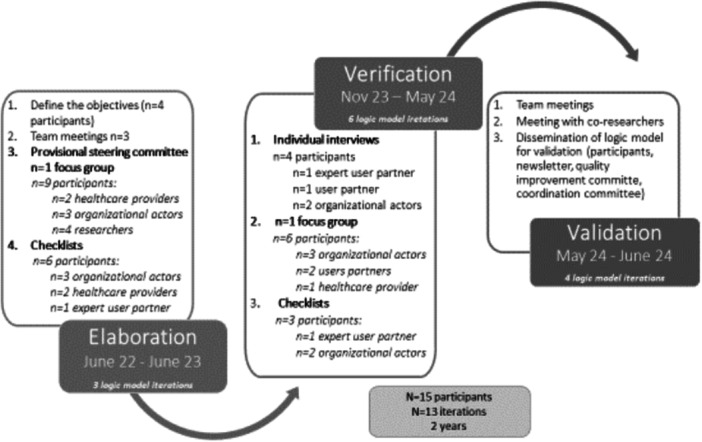
Sources and iterative process of the co‐creation of the HOPtimise logic model.

Among these 15 participants, the distribution of status and roles is shown in Table 1. All users and expert user partners have personal experience with paediatric cancer, either as former patients or as accompanying family members. The research team trained all the user partners in patient partnership, including those designated as experts with significant experience in research, program implementation or supporting families. As shown in Figure 1, three main sources of data were used: two focus groups, four individual interviews and nine checklists.

#### 2.2.2 Data Collection

The data collection method followed the Porteus [13] approach for building the logic model. According to the literature, the team followed a three‐step iterative data collection process: elaboration, verification and validation [15, 22, 24, 25]. The entire process—from the initial elaboration to finalisation and validation by researchers and user partners—took place from June 2022 to June 2024, and is summarised in Figure 1.

##### 2.2.2.1 Elaboration Phase

The elaboration phase included preparatory meetings. As Wholey et al. [28] recommend, the team first interviewed individuals closely associated with the program and its design, and then moved to those affected by the program and later to people involved as user partners or healthcare providers. The first meeting was with representatives of the Québec Ministry of Health and Social Services and researchers to reflect on the goals of HOPtimise, aiming to standardise HOP nursing practices and ensure equal access to quality care across Québec.

From this first phase, a draft emerged that was presented to the provisional steering quality improvement committee, through a focus group (September 2022) facilitated by the lead researcher and observed by two co‐investigators. The facilitation guide for the focus group was built on Porteus's structure of a logic model, collecting feedback and expertise on every component (mandatory and optional) of the logic model and asking participants for their opinions. Specifically, they were asked to provide feedback regarding the model's relevance to the context of the CHUL, possible missing elements and its clarity for different audiences.

Afterwards, the research team made the adjustments suggested by participants and sent a non‐mandatory checklist to all of them. Within a co‐design framework, this checklist's purpose was to demonstrate that feedback from the participants was incorporated and that the process was collaborative. It also showed that the corresponding adjustments have been made. Finally, it was a tool to determine whether the innovation was visually appealing and easily understandable.

##### 2.2.2.2 Verification Phase

For the second phase, the aim of the recruitment for individual interviews and a second focus group was to include additional user partners as well as strategic, organisational and operational actors involved in the project. The coordination committee assisted with recruitment of participants among their hospital contacts. The individual interviews were intended to gather in‐depth feedback on the logic model from participants involved in various committees, including the coordination committee, which oversees the implementation of the innovation. At the end of each interview, participants were asked to complete the checklist to add or correct elements after the research team made post‐interview adjustments. The focus group was designed to bring together stakeholders with different backgrounds to verify the relevance, feasibility and clarity of the logic model. The revised logic model was sent to participants along with the checklist, but none returned the checklist because they had nothing to add.

##### 2.2.2.3 Validation Phase

To validate the logic model, the lead researcher and co‐investigator verified that all the feedback had been successfully incorporated. The final phase involved disseminating the logic model to gather any additional feedback. The last iteration of the logic model was sent to all the participants, allowing them to suggest changes as needed, and to the quality improvement committee, as they are central to the innovation's elaboration and implementation. Finally, all stakeholders of the project received the logic model via a newsletter. The dissemination followed the strategies recommended by Porteus et al. (2002) for explaining a logic model to different audiences. A few minor adjustments were made to the logic model during the validation phase.

### 2.3 Analysis

The team gathered information from focus groups, interviews and checklists, and applied every modification suggested by participants using observations, live modifications, notes and recordings of the meetings. The deductive aspect of this process was based on the fact that a first draft of the logic model was created before data collection, and all the modifications were made step by step through each category and component of the logic model.

The analysis was also strengthened by creating of a visual and concise representation of the logic model, providing the team with a foundation for future evaluation of the elaboration phase and the innovation's implementation. The data collection process was documented in a diary maintained by the main investigators throughout the elaboration phase, confirming the iterative process and reinforcing the analysis with a chronological approach.

### 2.4 Criteria of Scientificity

Our methods align with best practices in qualitative research to ensure trustworthiness. First, credibility and confirmability are supported by the research team's extended involvement in the innovation project, participating in all meetings, which fostered consensus and minimised bias [29]. The participant panel (*n* = 15), mobilised through multiple sources of data (three different data collection methods), ensured data saturation [30]. This approach also supports reliability [24, 31] and dependability [29], which are also ensured by documenting all decisions made through the elaboration diary [26]. Finally, transferability was addressed by involving nurses from various organisations and haemato‐oncological services in Québec in the development of the logic model and the quality improvement committee. This is particularly important because the project aims for national deployment.

## 3 Results

**Figure 2:**
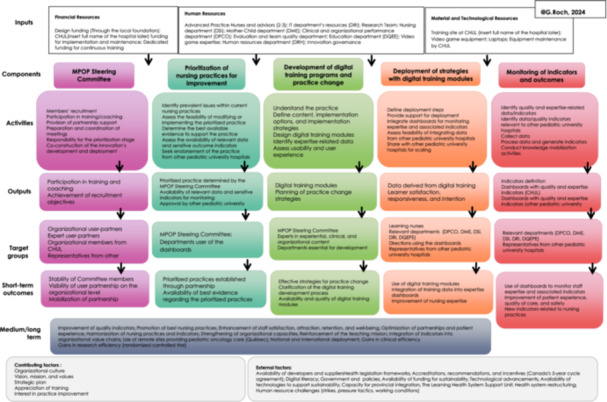
HOPtimise logic model. *All the departments are written entirely once in the logic model and then just named after initials.

The main results are presented in Figure 2, through the logic model of the innovation. However, the results also include all the elaboration and consolidation processes of this model, such as chronological observations and the roles and activities of the stakeholders.

### 3.1 Iterative Process and Chronological Observations

The elaboration phase was successful by allowing the team to develop an initial model that enabled recruitment and training of permanent quality improvement committee members. Having a strong initial logic model provided a foundation to present the innovation's goals, objectives, components, main activities and expected outcomes to committee members.

The first phase included researchers, organisational actors and healthcare providers who were central to the project. The purpose of this phase was to design the framework of the innovation and define the main elements related to the project context and its aims. In collaboration with internal and external actors, the team also aimed to identify the main goals and draft the inputs, outputs and outcomes.

Building on this initial phase, the verification phase allowed the inclusion of additional stakeholders in the co‐creation process of the logic model by recruiting more organisational actors and user partners. The aim was to (1) verify and strengthen the logic model, (2) gain professional insight from practitioners and actors experienced in implementing innovations at CHU de Québec and (3) involve user partners to make sure that patients' perspectives and experiences aligned with the innovation's goals and that the logic model was clear to all.

The final phase was successful, validating the logic model and disseminating it to all stakeholders in order to (1) provide them with the results and ensure that everyone had an understanding of the innovation and its components, and (2) offer opportunities for new ideas and recommendations. Ultimately, four iterations resulted from this phase.

### 3.2 Consolidation of the Roles and Activities of Stakeholders and Committees

From the beginning, many stakeholders were involved in the project. Nevertheless, initially, the research team was the main actor in putting the project in motion. The quality improvement steering committee is based on the patient and family engagement's principles according to Carman et al. [19] and Bombard et al. [20], and the users have all been successfully trained for patient and user partnership based on the recommendations of the Ministère de la santé et des services sociaux [32] (Cadre de références MSSS) during the elaboration phase of the logic model (starting 28 November 2022) by an expert user partner and a co‐researcher. The provisional quality improvement steering committee took place 2 months prior to training (September 2022). Accordingly, one should consider that the start of the second phase, the co‐creation of the logic model, enabled the transfer of certain prerogatives from the research team to the steering committee and to the coordination committee. These are the committees centrally involved in the co‐creation of the innovation and its implementation through the first two components of the logic model, specifically, the quality improvement committee and the prioritisation of practices. They have related activities such as participation in training or responsibility for the prioritisation stage. Some of the main expected outcomes from establishing a stable steering quality improvement committee were the visibility of user partnership on the organisational level and a prioritisation of practices carried out through this partnership.

The coordination committee oversees the implementation of the innovation by making sure that the progress and operationalisation of the project are satisfactory. In the logic model, members are represented in two ways: within human resources, via committees and services, and within the components, as organisational members. Creation of a logic model made its purpose more tangible by communicating through the governance's different committees and helped stabilise the members' recruitment and implication in the project.

The logic model (Figure 2) presents the causal links among the inputs, the main components, the more detailed activities, the outputs, the targeted audiences and the short‐ and mid‐/long‐term expected results.

The aim of the innovation is to, with user partners, improve nursing practices in paediatric haemato‐oncological services in Quebec by creating training modules that enable the development of dashboards containing indicators of quality and expertise sensitive to the specificity of nursing practices. This goal plainly manifests itself in what Wholey et al. [28] call the ‘Results from program’ and corresponds to the outcomes, either short‐term outcoles of medium‐long outcomed [13]. Notably, the outcimes represent the ‘Why’ of the innovation and so, the purpose of the logic model.

The following are the subsequent aims of the innovation:

- Create a user partnership to improve the quality of care and services by prioritising best practices with patient partners.
- Elaborate and develop training modules that will be included in a serious game to support best nursing practices.
- Highlight sensitive indicators of quality and expertise in nursing practices.

The prioritisation of best practices, in co‐design with user partners, intends to consolidate the nursing practices by creating training modules, including digital training (*serious game*), allowing the generation of dashboards for sensitive quality and expertise indicators.

### 3.3 Clarifying the Purposes of the Innovation

The collaborative creation of the logic model enabled the stakeholders to co‐design their involvement in the elaboration and implementation of the innovation. It also provided the user partners with a clear idea of the main expected outcomes and the activities that they would take part in, based on their experiences and expertise, especially within the quality improvement committee, for instance, in the *mobilisation of partnership* in the short term and *optimisation of partnerships and patient experience* in the medium/long term. Communication paled a key role in the iterative process by integrating the thoughts and expertise of everyone and then disseminating the logic model to all other stakeholders.

The process also enabled everyone involved to see the internal logic of the project and to understand the relationship between the activities and the expected outcomes, as well as to assess how the components of the innovation would impact the target audience. For instance, the quality improvement committee has a fundamental responsibility in the prioritisation of nursing behaviour (as listed in the ‘activities’ section). Then—according to the output section—those practices are *established through partnership* and the *availability of best evidence* and so, they are prioritised. These steps can lead, for example, to the *integration of training data into expertise dashboards* sustained by *experts in experiential and clinical content and department*.

### 3.4 Evaluation of the Results and Outcomes

The elaboration of the logic model generated extensive discussions about the expected outcomes. The team proposed outcomes aligned with the project's main objectives and identification of short‐, intermediate‐ and long‐term outcomes. The verification phase of the logic model highlighted the importance of having clear expected outcomes and measurable indicators, providing reassurance to stakeholders. In interviews, focus group and checklists, concerns about the innovation's feasibility and the identification and evaluation of real outcomes for patients and on the target audience (nurses) were central. Are the outcomes measurable? Can the outcomes be prioritised by importance? Is the innovation feasible and sustainable? Those were the main questions regarding the outcomes. These issues were especially raised by organisational actors responsible for implementing the innovation within their areas. Although the research team initially did not plan to include such detailed outcomes in the logic model, analysis indicated that it was essential to stakeholders. This process heightened the team's awareness of the need to clarify causal links and prompted discussions about the materials, experiences and expertise required to support the logic model's causal relationships. Additionally, the intention of creating dashboards for quality indicators generated feedback. Organisational actors requested more details on dashboards, as their complexity and goals needed clarification, leading the team to specify in each component where dashboards would be relevant. Participants also noted—in the outputs—the lack of clarity regarding the innovation's deployment, implementation sites and the role of the other medical facilities in prioritising practices. The team made adjustments by specifying additional sites beyond CHU de Québec for deployment, and regarding provincial and national deployment. However, no changes were made regarding prioritisation, as actors from other sites were already included in governance and in the prioritisation process. However, this feedback led to further reflection on how the administration of other sites' administrations would adapt training decisions to their own structure and needs.

### 3.5 Questioning the Resources

Adjustments were made based on feedback regarding human resources in the input section. The analysis enabled greater specificity about the various directions of the project and committees involved in the project, improving understanding of how actors will participate in different activities by listing them directly—not only in the input section but also in the targeted groups. Human resources were a significant concern from the start of the iterative process. The input section raised questions on whether available human resources would be sufficient—not just to carry out the planned activities in the logic model but also to sustain them over time.

Other considerations were raised. The paediatric oncological service is small, and the targeted audience is limited (there will be 33 nurses using the digital training). In addition, there are challenges related to human resources movement within the service, prompting discussions about the availability of nursing teams. On the one hand, there are concerns that there may not be enough advanced practice nurses to provide expertise for creating the training modules; on the other hand, regarding feasibility and resources, the team must find time for nurses in training to use the serious game during their working hours.

### 3.6 Contributing and External Factors

As an innovation aiming both to standardise practices through digital training (serious game) and to adapt training across different provincial sites (improving best practices beyond the serious game), it was essential to identify the factors that could influence deployment at other sites. Collaborative efforts focused on contributing and external factors that would affect the success of achieving expected outcomes. In general, the involvement of various stakeholders, either internal or external to the CHU de Québec's structure, was transversal to all the components of the logic model and an important concern for the participants. Development of the logic model allowed identification of factors within the program, such as vision, mission, values and organisational culture, which were seen as a fertile environment for implementation of innovation. Reflections on contributing and external factors highlighted approaches for resource sustainability, involvement of different actors, data accessibility and deployment strategies. Although Porteus [13] presents these factors as optional in visual logic models, their inclusion enriched the logic model, reinforced causal links between components and set the stage for organisational reflection and future strategies.

## 4 Discussion

### 4.1 Summary of Findings

As HOPtimise is a complex innovation, having a visual representation of its components was necessary for the sake of the whole stakeholders' understanding. The diverse profiles and backgrounds of stakeholders required a clear and concise visual that could provide a shared understanding from any actor—user partners, organisational actors, strategic actors, healthcare providers, external managers, researchers, funders and administrators [12, 15]. The objectives of developing a logic model were achieved through a 2‐year participatory process, resulting in 13 iterations and consensus‐based decision‐making [21]. All stakeholders involved in the elaboration of the intervention are considered as equal and complementary to the research team members [33]. The thorough integration of the stakeholders' expertise and experiences throughout this sustained iterative process offers guidance for similar complex organisational innovations, particularly those with small target audiences and/or a niche sector. The purpose is also to ‘close the gap between academic knowledge production and its practical use by communities’ [33, p. 2]. The co‐creation of the logic model can serve both as a model for improving patient and public contribution in organisational innovation research and as a guide for future health improvement interventions in nursing practices. The micro‐dimension of this intervention made it challenging to find adequate expertise. This is why it implies a very thorough and complete logic model and multiple perspectives. The three research phases mobilised various governance resources—expertise, experience, doubts and feedback—to enrich the logic model. The two focus groups specifically provided a strong consensus [12], interviews allowed the team to explore stakeholder concerns in depth and checklists reinforced information gathered previously.

Initially considered too complex and lacking clarity by some actors, the logic model was transformed into a visual program to provide a common vision for all aspects of the innovation's development and implementation. This inclusive approach—engaging all types of actors and using multiple data collection methods—helped prevent negative power dynamics and provided time for reflection, making stakeholders feel involved. It also established a foundation for including stakeholders in activities that they would lead or participate in, and supporting partnership and patient engagement. It generated a healthy environment for communication through the different committees that would be created over the following months. As Bisson et al. [15], citing Danish et al. [34], note that generating patient engagement early in the program helps ensure that stakeholders maintain a shared understanding of the project. The logic model reveals the participation of user partners (in the quality improvement committee for instance) and of various service departments, but there is still lack of clarity about specific stakeholder roles within the five components. Danish et al. [34] suggest focusing on resources, processes and relationships, and the HOPtimise logic model could have benefited from greater emphasis on relationships. The logic model was shared with all actors and now serves as a strong communication tool for every meeting with new participants, partners and institutions.

During the first year of the iterative process, the logic model became robust enough to support an efficient start for several key activities, such as finalising the recruitment and training of user partners for the quality improvement committee, and conducting the first and second rounds of best practices prioritisation for training modules to be included in the serious game scenario. Indeed, between Phase 1 and Phase 2, the team collaborated with healthcare providers and the quality improvement committee to define practice change strategies within the haemato‐oncological service. In Phase 2, a partnership was established with the game company, developing most of the serious game assets through collaborative work. Finally, during the validation phase, from May to August 2024, the logic model was refined enough for the research team to begin individual interviews with strategic and organisational actors in order to identify strategies for overcoming potential barriers. Emerging facilitators and barriers were discussed with key organisational actors, and, through the CFIR guide and ERIC‐matching tool [35, 36], the team developed an Implementation research logic model [37] to map strategies.

Disseminating the logic model to the stakeholders facilitated the transfer of competencies from the research team to the organisational actors. The research team continues to evaluate each major step and the outcomes presented by the logic model. For example, partnership and patient experience are being optimised through various data collections—either within the quality improvement committee or by developing evaluation guides for the experience of patients in collaboration with the CHU de Québec audit team.

Patient engagement is one of the pillars of the innovation, and establishing a quality improvement committee led by patients and caregivers is a primary goal [19]. The intent of this change‐oriented project is to have patients at the centre of the decision‐making process, and the elaboration of the logic model demonstrates that integrating patients serves an organisational purpose. This is why the study fulfils the aim of a participatory approach by empowering patients towards a collaborative organisational innovation [23].

### 4.2 Limitations

As participants expressed concerns towards feasibility because of the resources involved and the challenges behind quality indicators, and even if the goals and objectives of the innovation were already presented, the team could have included a more explicit goal and objective section in the logic model. It would have probably improved the understanding of the causality between goals, activities, outputs and outcomes. Fuller et al. [12] included goals in their logic model, making the logic more understandable. Additionally, the current logic model does not adequately reflect the presence of the coordination committee, as noted in recent checklists. The committee should be presented directly in the logic model, not just as an implicit result of the logic model's creation.

Due to the complexity of the innovation, it was challenging to create direct links between the desired outcomes and the related activities. The team could have included a timeline in the logic model to provide a perspective on feasibility. However, given the five components and the series of activities, it was difficult to visually relate the various terms—especially short‐ and intermediate‐term outcomes—to specific activities. The logic model needed to be understandable for all audiences and be visually appealing without being overcrowded. Several participants remarked that it was already quite busy.

Finally, the small targeted audience (33 nurses) made it difficult to accurately evaluate resource management needs and to create time for advanced practice nurses to participate in developing the serious game. This is why inclusion of more nurses in the elaboration of the logic model would have provided more relevant and practical insights on inputs and on the overall program. Of the 15 participants, only four were nurses. The initial implementation phases revealed that time and human resources remain important concerns, prompting reflection on incentives and allowances.

## 5 Conclusion

This study presented the three‐phases iterative development process of the logic model of HOPtimise, a paediatric oncology innovation aimed at improving nursing practices in the province of Quebec, Canada. Over 2 years, a 15‐multistakeholder team—including organisational and user partners—participated in numerous iterations of the logic model through elaboration, verification and validation, following the Porteus et al. (2009) model. The logic model has been proven to be a successful tool for clarifying and communicating among stakeholders. Development of the logic model also led to the creation of a permanent quality improvement committee. Having a solid logic model enabled the construction of a research logic model and identification of the best implementation strategies, as well as barriers and facilitators. Finally, the logic model currently remains a supportive tool for researchers to keep track of the project, for the creation of evaluation tools of the innovation and its implementation, and for the expected activities and their outcomes.

## Author Contributions

**Martin Blanc:** conceptualisation, writing – original draft, data curation, methodology, writing – review and editing, formal analysis. **Marie‐Pierre Gagnon:** conceptualisation, supervision, writing – review and editing. **Daphney St‐Germain:** validation, supervision, writing – review and editing. **Boutheina Mejri:** investigation, writing – review and editing. **Marie Gagné:** resources. **Isabel Bean:** project administration. **Gratianne Vaisson:** writing – review and editing.

## Disclosure

The views expressed in this publication and the analysis are those of the authors of the article and not of the CHUL hospital or Charles Bruneau foundation.

## Ethics Statement

Ethical approval was granted by the Bureau de l'éthique de la recherche, CHU de Québec‐Université Laval and Centre de recherche CHU de Québec‐Université Laval (Ref: 2022‐5899‐21).

## Consent

Informed consent was obtained from all participants prior to focus groups and interviews.

## Conflicts of Interest

The authors declare no conflicts of interest.

## Data Availability

The data that support the findings of this study are available on request from the corresponding author. The data are not publicly available due to privacy or ethical restrictions.

## References

[1] Comité consultatif des statistiques canadiennes sur le cancer, Statistiques canadiennes sur le cancer 2019 (Société canadienne du cancer, 2019).

[2] L. A. Linder and J. Challinor , “Pediatric Oncology Nurse‐Led Research and Evidence‐Based Practice: Global Exemplar to Reduce Disparity,” Journal of Pediatric Hematology/Oncology Nursing 40, no. 5 (2023): 281–285. 37971201 10.1177/27527530231190371

[3] CureAll Framework: WHO Global Initiative for Childhood Cancer. Increasing Access, Advancing Quality, Saving Lives (World Health Organization, 2021).

[4] E. A. H. Loeffen , L. C. M. Kremer , R. L. Mulder , A. Font‐Gonzalez , L. L. Dupuis , et al., “The Importance of Evidence‐Based Supportive Care Practice Guidelines in Childhood Cancer—A Plea for Their Development and Implementation,” Supportive Care in Cancer 25, no. 4 (2017): 1121–1125. 27928642 10.1007/s00520-016-3501-y PMC5321691

[5] B. Love , “Baccalaureate‐Linked Oncology Nursing Education: McMaster University's Paediatric and Adult Oncology Nursing Program,” Canadian Oncology Nursing Journal/Revue canadienne de soins infirmiers en oncologie 15, no. 2 (2005): 80–86. 10.5737/1181912x1528086 15969331

[6] M. G. Meraviglia , C. McGuire , and D. A. Chesley , “Nurses' Needs for Education on Cancer and End‐of‐Life Care,” Journal of Continuing Education in Nursing 34, no. 3 (2003): 122–127. 12772811 10.3928/0022-0124-20030501-08

[7] N. M. E. Bradley , P. D. Robinson , M. L. Greenberg , R. D. Barr , et al., “Measuring the Quality of a Choldhood Cancer Care Delivery System: Assessing Stakeholder Agreement,” Value in Health 16, no. 4 (2013): 639–646. 23796299 10.1016/j.jval.2013.02.016

[8] C. E. Sullivan , S. W. Day , N. Ivankova , A. Markaki , P. A. Patrician , and W. Landier , “Establishing Nursing‐Sentive Quality Indicators for Pediatric Oncology: An International Mixed Methods Delphi Study,” Journal of Nursing Scholarship 55, no. 1 (2022): 388–400. 35790072 10.1111/jnu.12798 PMC9946155

[9] A. Van Driessche , J. Gilissen , A. De Vleminck , M. Kars , and J. Fahner , “The BOOST Paeditruc Advance Care Planning Intervention for Adolescents With Cancer and Their Parents: Development, Acceptability and Feasibility,” BMC Pediatrics 22 (2022): 210. 35428281 10.1186/s12887-022-03247-9 PMC9010242

[10] C. Balice‐Bourgois , C. J. Newman , G. D. Simonetti , and M. Zumstein‐Shaha , “A Comple Interprofessional Intervention to Improve the Management of Painful Procedures in Neonates,” Paediatric & Neonatal Pain 2, no. 3 (2020): 63–73. 35547023 10.1002/pne2.12012 PMC8975212

[11] The Hospital for Sick Children (SickKids), University of Toronto, 2006‐2026, Training program. https://www.sickkidsinternational.ca/.

[12] L. Fuller , J. Beattie , V. Ramsbottom , B. Condon , et al., “Developing a Program Logic Model for Evaluation and Research of a Rural Medical Training Stream,” BMC Medical Education 25 (2025): 749. 40405266 10.1186/s12909-025-07315-3 PMC12096687

[13] N. L. Porteus , “La construction du modèle logique d'un programme,” dans Approches et pratiques en évaluation de programme, (dir.) Ridde, V. and Dagenais C. (Les presses de l'université de Montréal, 2009), 87–106.

[14] C. Jinks , P. Carter , C. Rhodes , et al., “Patient and Public Involvement in Primary Care Research ‐ An Example of Ensuring Its Sustainability,” Research Involvement and Engagement 2 (2016): 12, 10.1186/s40900-016-0015-1. 29062502 PMC5611572

[15] M. Bisson , K. Aubrey‐Bassler , M.‐C. Chouinard , S. Doucet , V. R. Ramsden , et al., “Patient Engagement in Health Implementation Research: A Logic Model,” Health Expectations 26 (2023): 1854–1862. 37309078 10.1111/hex.13782 PMC10485341

[16] S. Michie , M. M. van Stralen , and R. West , “The Behaviour Change Wheel: A new Method for Characterising and Designing Behaviour Change Interventions,” Implementation Science 6 (2011): 11, http://www.implementationscience.com/content/6/1/42. 21513547 10.1186/1748-5908-6-42 PMC3096582

[17] S. G. Marble , “Five‐Step Model of Professional Excellence,” Clinical Journal of Oncology Nursing 13, no. 3 (2009): 310–315. 19502189 10.1188/09.CJON.310-315

[18] J. D. Kirkpatrick and W. K. Kirkpatrick , Kirkpatrick's Four Levels of Training Evaluation. BusinessPro Collection (ATD Press, 2016).

[19] K. L. Carman , P. Dardess , M. Maurer , et al., “Patient and Family Engagement; a Framework For Understanding The Elements and Developing Interventions and Policies,” Health Affairs 32, no. 2 (2013): 223–231. 23381514 10.1377/hlthaff.2012.1133

[20] Y. Bombard , G. R. Baker , E. Orlando , et al., “Engaging Patients to Improve Quality of Care: A Systematic Review,” Implementation Science 13 (2018): 1–22. 30045735 10.1186/s13012-018-0784-z PMC6060529

[21] K. Pajar , C. Honeywell , H. Howley , N. Sheridan , W. Affleck , et al., “Participatory Logic Model for a Precision Child and Youth Mental Health Start‐Up: Scoping Review, Case Study, and Lessons Learned,” Frontiers in Health Services 4 (2024): 1405426. 39483443 10.3389/frhs.2024.1405426 PMC11524936

[22] M.‐D. Primeau , C. Clausen , and M. Lavoie‐Tremblay , “Cocréation d'un modèle logique dans une démarche d'amélioration continue: le cas du Strengths‐Based Nursing,” Revue francophone internationale de recherche infirmière 9 (2023): 100307, 10.1016/j.refiri.2023.100307.

[23] J. W. Creswell , Research Design: Qualitative, Quantitative, and Mixed‐Methods Approaches. 3rd ed. (Sage, 2009).

[24] P. Fusch , G. E. Fusch , and L. R. Ness , “Denzin's Paradigm Shift: Revisiting Triangulation in Qualitative Research,” Journal of Social Change 10, no. 1 (2018): 19–32.

[25] N. K. Denzin , The Research at: A Theoretical Introduction to Sociological Methods. 2nd ed. (McGraw Hill, 1978).

[26] C. Baribeau , “Le journal de bord,” Recherches qualitatives, Hors‐série 2 (2005): 98–113.

[27] A.‐A. Houle , A. Dupuis , P. Dionne , J. Lane , and D. Therriault , “‘L'apport du journal de bord en évaluation de programmes dans une perspective de pérennisation: le cas du programme HORS‐PISTE’. Évaluation de programme,” Canadian Journal of Program Evaluation/La Revue Canadienne d'évaluation de programme 36, no. 1 (2021): 43–63.

[28] J. Wholey , H. P. Hatry , and K. E. Newcomer , Handbook of Practical Program Evaluation. 2nd ed. (Jossey‐Bass. A Wiley Imprint. Indiana University, 2004).

[29] S. K. Ahmed , “The Pillars of Trustworthiness in Qualitative Research,” Journal of Medicine, Surgery, and Public Health 2 (2024): 100051, 10.1016/j.glmedi.2024.100051.

[30] P. Fusch and L. Ness , “Are We There Yet? Data Saturation in Qualitative Research,” Qualitative Report 20 (2015): 1408–1416.

[31] C. Stavros and K. Westberg , “Using Triangulation and Multiple Case Studies to Advance Relationship Marketing Theory,” Qualitative Market Research 12 (2009): 307–320.

[32] E. Fournier , S. Veilleux , Cadre de référence de l'approche de partenariat entre les usagers, leurs proches et les acteurs en santé et en service sociaux. Gouvernement du Québec, 2018). https://publications.msss.gouv.qc.ca/msss/fichiers/2018/18-727-01W.pd.

[33] J. Cumming , R. Whiting , B. J. Parry , et al., “The My Strengths Training for Life Program: Rationale, Logic Model, and Description of a Strengths‐Based Intervention for Young People Experiencing Homelessness,” Evaluation and Program Planning 91 (2022): 12p. 10.1016/j.evalprogplan.2021.102045 35032787

[34] A. Danish , M. Karam , J. Porter , et al., “Planning the Evaluation of PatientEngagement in the PriCare Research Program,” Canadian Journal of Program Evaluation 37, no. 1 (2022): 104–116.

[35] L. J. Damschroder , C. M. Reardon , M. A. O. Widerquist , and J. Lowery , “The Updated Consolidated Framework for Implementation Research Based on User Feedback,” Implementation Science 17, no. 1 (2022): 75, 10.1186/s13012-022-01245-0. 36309746 PMC9617234

[36] L. J. Damschroder , D. C. Aron , R. E. Keith , S. R. Kirsh , J. A. Alexander , and J. C. Lowery , “Fostering Implementation of Health Services Research Findings Into Practice: A Consolidated Framework For Advancing Implementation Science,” Implement Science 4, no. 1 (2009): 50. 10.1186/1748-5908-4-50 PMC2736161 19664226

[37] A. A. Knapp , A. J. Carroll , N. Mohanty , et al., “A Stakeholder‐Driven Method for Selecting Implementation Strategies: A Case Example of Pediatric Hypertension Clinical Practice Guideline Implementation,” Implementation Science Communications 3, no. 1 (2022): 25. 35256017 10.1186/s43058-022-00276-4 PMC8900435

